# Size-dependent cellular uptake mechanism and cytotoxicity toward calcium oxalate on Vero cells

**DOI:** 10.1038/srep41949

**Published:** 2017-02-02

**Authors:** Xin-Yuan Sun, Qiong-Zhi Gan, Jian-Ming Ouyang

**Affiliations:** 1 Department of Chemistry, Jinan University, Guangzhou 510632, China; Institute of Biomineralization and Lithiasis Research, Jinan University, Guangzhou 510632, China

## Abstract

Urinary crystals with various sizes are present in healthy individuals and patients with kidney stone; however, the cellular uptake mechanism of calcium oxalate of various sizes has not been elucidated. This study aims to compare the internalization of nano-/micron-sized (50 nm, 100 nm, and 1 μm) calcium oxalate monohydrate (COM) and dihydrate (COD) crystals in African green monkey renal epithelial (Vero) cells. The internalization and adhesion of COM and COD crystals to Vero cells were enhanced with decreasing crystal size. Cell death rate was positively related to the amount of adhered and internalized crystals and exhibited higher correlation with internalization than that with adhesion. Vero cells mainly internalized nano-sized COM and COD crystals through clathrin-mediated pathways as well as micron-sized crystals through macropinocytosis. The internalized COM and COD crystals were distributed in the lysosomes and destroyed lysosomal integrity to some extent. The results of this study indicated that the size of crystal affected cellular uptake mechanism, and may provide an enlightenment for finding potential inhibitors of crystal uptake, thereby decreasing cell injury and the occurrence of kidney stones.

Hyperoxaluria is a well-recognized risk factor for urolithiasis; patients with primary hyperoxaluria gradually develop calcium oxalate (CaOx) deposits, as well as causing renal tubule damage directly via oxalate toxicity^1^,^2^. CaOx is a main component of urinary calculi. CaOx crystals adhere to the renal tubular epithelial cells and deposit into the renal tubular lumen and interstitium, resulting in tissue injury and dysfunction^3^,^4^. Adhesion between the crystals and the cells is the early process of stone formation^5^, and the adherent crystals can be internalized by cells, leading to serious injury^6^. Cells can endocytose calcium oxalate monohydrate (COM) crystals. For example, kidney epithelial cells in monolayer culture (BSC-1 line) rapidly bind and internalize COM crystals, which dissolve within lysosomal inclusion bodies from 5 to 7 weeks^7^. Kanlaya *et al*.^8^ found that MDCK cells endocytose COM crystals with a size of 3–5 μm mainly through macropinocytosis.

The pathway of cellular endocytosis is influenced by particle size, morphology, and surface charge. Hao *et al*.^9^ reported that spherical mesoporous silica (SiO_2_) nanoparticles are internalized via the clathrin-mediated pathway; SiO_2_ particles with high aspect ratios (aspect ratio = 4) are internalized through the caveola-mediated pathway. Endocytosis of negatively charged nanoparticles in cells is slower than that of positively charged nanoparticles because of the negative charge of the cell membrane. However, the endocytosis rate of negatively charged quantum dot nanoparticles is higher than that of neutral or positively charged quantum dots^10^. Mostly, particles with size >5 μm are mainly endocytosed through macropinocytosis and phagocytosis; moreover, nanosized crystals are endocytosed through the clathrin-mediated endocytosis pathway^11^.

The sizes, crystal phases, and size distribution of urinary crystals significantly differ between healthy individuals and patients with kidney stones^12^,^13^. COM and calcium oxalate dihydrate (COD) crystals with various sizes induce varied degrees of cytotoxicity and cellular responses^14^,^15^. However, the size effect of nano-/micron-sized COM and COD crystals on cellular internalization in kidney epithelial cells has not been reported yet. Vero cells isolated from kidney epithelial cells of an African green monkey are one of the most commonly used mammalian continuous cell lines in research on kidney stones^16^,^17^. Thus, in the present study, COM and COD crystals of different sizes (50 nm, 100 nm, and 1 μm) were prepared and compared in terms of endocytosis pathway and internalization mechanism toward Vero cells to reveal the cytotoxicity mechanism of kidney stone formation.

## Results and Discussion

### Fluorescently labeled nano-/micron-sized COM and COD crystals

**Figure 1:**
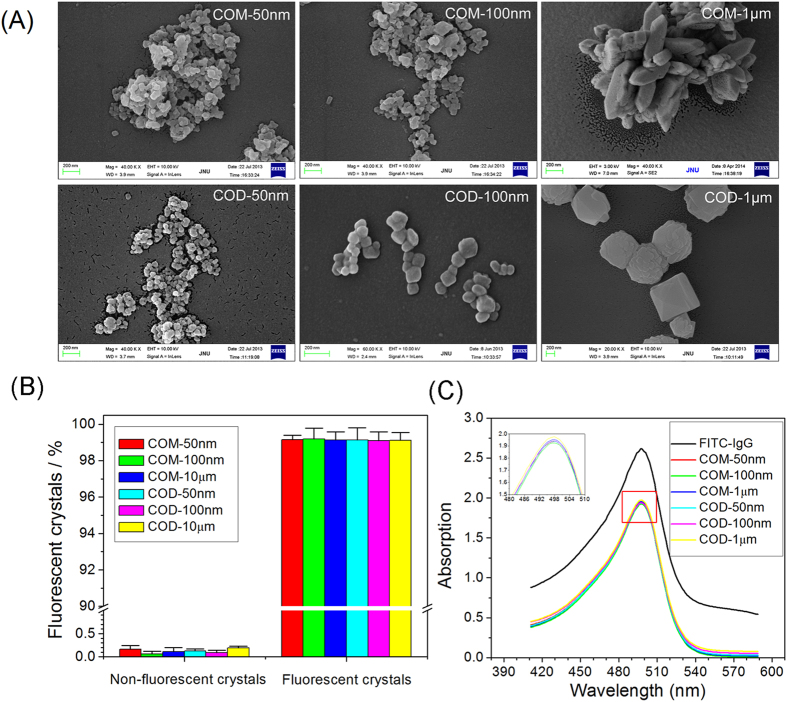
Characterization of nano-/micron-sized COM and COD crystals. (**A**) Morphological observation of nano-/micron-sized COM and COD crystals. (**B**) Percentages of fluorescent COM and COD crystals detected by flow cytometry analysis. More than 99% of FITC–IgG-conjugated crystals were detected as fluorescent crystals, and the background of the nonfluorescent crystals was negligible. (**C**) Absorbance of FITC before and after labeling with nano-/micron-sized COM and COD crystals.

Figure 1A shows the SEM images of the prepared nano-/micron-sized COM and COD crystals. The sizes of the COM and COD crystals are 50 nm, 100 nm, and 1 μm. We used an integer (COM-50 nm, COM-100 nm, COM-1 μm, COD-50 nm, COD-100 nm and COD-1 μm) to represent the crystal size for simplicity and convenience. The crystal phase was detected by XRD and FT-IR characterization presented in our previous study^18^. All the prepared samples are pure-phase COM or COD crystals.

COM and COD crystals were labeled with FITC–IgG and showed green fluorescence under a fluorescence microscope^19^. The morphology of fluorescently labeled crystals is consistent with that of the unlabeled crystals. Statistical analysis of fluorescently labeled COM and COD crystals showed that more than 99% of the crystals were labeled as fluorescent (Fig. 1B). The spectra of FITC–IgG-labeled COM and COD crystals are characterized by UV-Vis spectroscopy. The FITC characteristic absorption peak was observed at 488–500 nm, and absorption intensity was determined (Fig. 1C). CaOx crystals were labeled with FITC–IgG, and the six crystals exhibited similar fluorescence intensities. These results demonstrated that fluorescently labeled COM and COD can be detected by flow cytometry and used for quantitative analysis.

### Adhesion of CaOx to Vero cells

Vero cells were incubated with the crystals at 4 °C, at which endocytosis is hindered but adhesion is unaffected^20^. Hovda *et al*.^6^ confirmed that binding CaOx to kidney epithelial cells reached the maximum within several minutes. Numerous studies adopted 1 h incubation in crystal adhesion assay to produce maximally saturated binding^21^,^22^. Thus, the cells were exposed to three sizes of fluorescence-labeled COM and COD crystals and then incubated at 4 °C for 1 h. The percentage of cells with adhered crystals was quantitatively determined through flow cytometry. Cells with positive FITC signals were directly counted and considered as containing adherent crystals.

**Figure 2:**
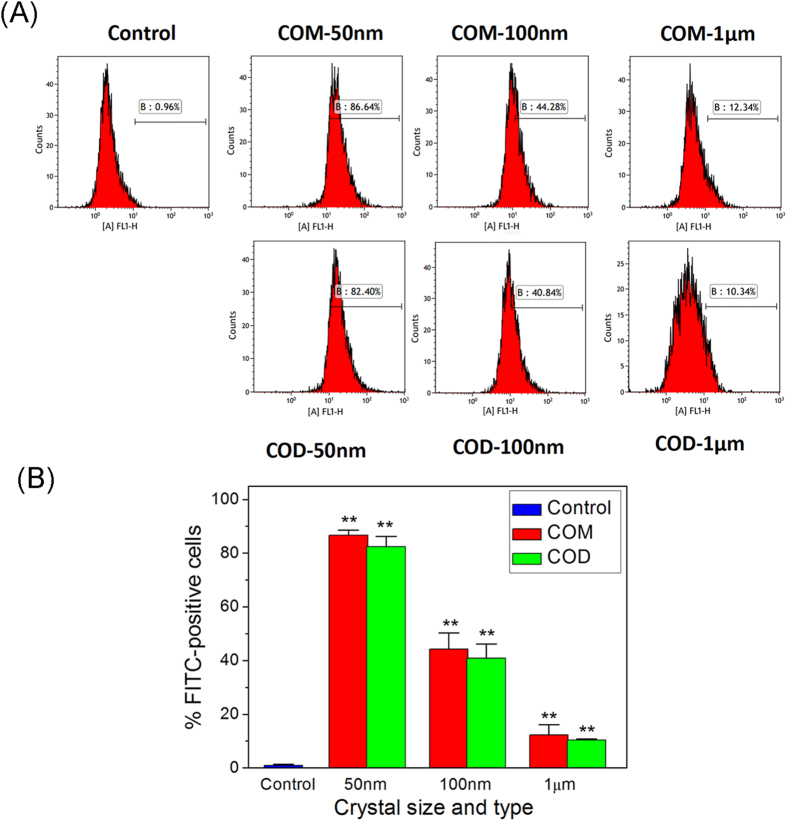
Flow cytometry quantitative analysis of adherent nano-/micron-sized COM and COD crystals. (**A**) Mean fluorescence intensity (FITC-A) and fluorescence ratio of cells. (**B**) Statistical analysis of the percentage of FITC-positive cells (cells adhered crystals). Compared with the control group, *p < 0.05, **p < 0.01.

The percentage of cells with adhered crystals decreased with increasing crystal sizes in each COM-treated group (Fig. 2A). Chutipongtanate *et al*.^23^ showed that approximately 6% of MDCK cells adhered to COM crystals (crystal size of approximately 10 μm) at 100 μg/mL. In the present study, the percentage of cells that adhered to COM-50 nm, COM-100 nm, and COM-1 μm reached 86.64%, 44.28%, and 12.34%, respectively. This phenomenon may be due to several factors: 1) small-sized crystals exhibit higher surface area than that of large-sized crystals and follow the order of COM-50 nm (26.3 m^2^/g) > COM-100 nm (14.7 m^2^/g) > COM-1 μm (13.6 m^2^/g) > COM-10 μm (0.83 m^2^/g)^18^; hence, crystals with high surface area can create abundant active sites for cell adherence; 2) number of small-sized crystals is higher than that of large-sized crystals under the same crystal concentration; 3) large-sized COM crystals cannot completely form a contact with the cell interface, resulting in reduced shear stress and weak adhesive force between the cells and the large-sized crystals^24^.

The number of cells that adhered to crystals in COD-treated groups is similar to that in COM-treated groups (Fig. 2A). Cell adherence with crystals followed the order of COD-50 nm (82.40%) > COD-100 nm (40.84%) > COD-1 μm (10.34%). Small crystal sizes reflect a high degree of crystal adhesion in Vero cells.

The adhesion to Vero cells is not significantly different between the COM and the COD crystals because of similar surface areas of COM and COD crystals with similar sizes. Wang *et al*.^25^ reported that HK-2 cells adhere to COM and COD crystals (crystal size of approximately 10 μm) without evident difference in quantity.

### Internalization pathways of CaOx with different sizes

Endocytosis is a process through which eukaryotic cells internalized extracellular substances through membrane deformation^26^. Endocytosis is mainly categorized as energy independent and dependent. The latter involves four distinct pathways^27^, namely, clathrin-mediated endocytosis, caveola-mediated endocytosis, macropinocytosis, and phagocytosis. Phagocytosis primarily occurs in specific phagocytes, such as macrophages, monocytes, and dendritic cells^28^. This pathway is not considered in this paper because Vero cells are normal kidney epithelial cells.

**Figure 3:**
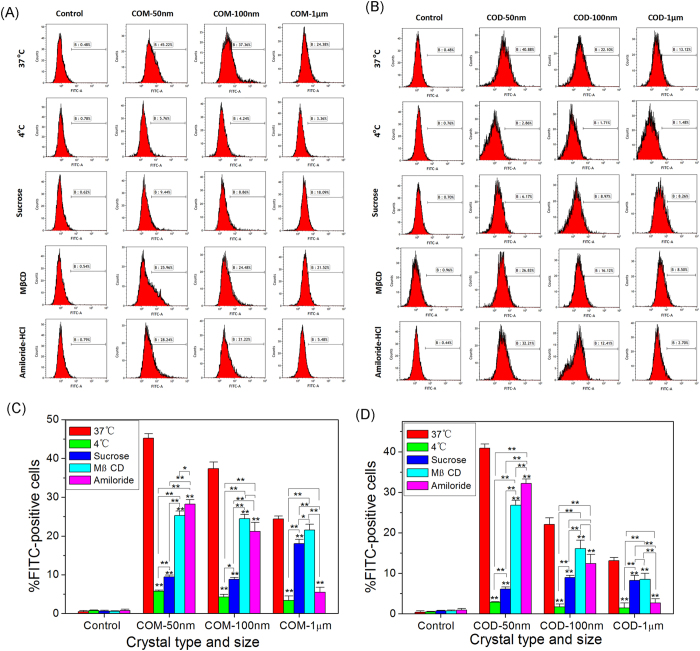
Flow cytometry quantitative analysis of internalized nano-/micron-sized crystals. (**A**,**C**) COM treatment groups and (**B**,**D**) COD treatment groups. Mean fluorescence (FITC-A) and fluorescence ratio of cells (**A**,**B**), whereas statistical analysis of the percentage of FITC-positive cells (**C**,**D**). Vero cells were pretreated with different endocytosis inhibitors at 37 °C for 30 min; clathrin-mediated endocytosis, 200 mM sucrose; caveola-mediated endocytosis, 10 mM methyl-β-cyclodextrin; and macropinocytosis, 1 mM amiloride hydrochloride. Compared with the corresponding normal internalization group at 37 °C, *p < 0.05, **p < 0.01.

The specific endocytosis pathways of nano-/micron-sized COM and COD absorbed by Vero cells were investigated in this study. The cells were pretreated with specific endocytosis inhibitors, such as sucrose, methy-β-cyclodextrin (MβCD), and amiloride hydrochloride, which correspond to clathrin-mediated endocytosis, caveola-mediated endocytosis, and macropinocytosis^29^,^30^. To analyze the energy-dependent endocytosis pathway, we used 4 °C for the treatment because cells consume less ATP and block the active transport at this temperature. A small number of Vero cells internalized nano-/micron-sized COM and COD at 4 °C, indicating the energy dependence of the internalization process (Fig. 3A and B).

Hovda *et al*.^6^ detected the internalization of CaOx crystals by using four types of proximal tubule (PT) cells. PT cells (HPT and RPT from Wistar and F344 rats) were exposed to 0.5 mL of [^14^C]-COM (74 μg/mL, 147 μg/mL, 294 μg/mL, or 440 μg/mL) for 0.5–8 h at 37 °C. When the exposure time reached 6 h, the internalization amount reached maximally saturated internalization. Thus, 6 h incubation was performed in the present study to detect amount of internalized crystals. About 45.22% and 40.88% of the cells internalized crystals in COM-50 nm and COD-50 nm treatment groups without inhibitors, respectively; the number was significantly reduced by treatment with sucrose (*p* < 0.01), followed by MβCD and amiloride hydrochloride (*p* < 0.01, Fig. 3). These results demonstrated that the COM-50 nm and COD-50 nm crystals internalized into the cells mainly through the clathrin-mediated pathway followed by the caveola-mediated and macropinocytosis pathways.

For COM-100 nm and COD-100 nm, the number of cells that internalized crystals significantly decreased by treatment with sucrose (*p* < 0.01) and decreased by a lesser degree in treatment with amiloride hydrochloride and MβCD (*p* < 0.01). Crystals with a size of 100 nm were also internalized into the cells mainly via the clathrin-mediated pathway, and some crystals were internalized by caveola-mediated and macropinocytosis pathway; these patterns are similar to those of 50 nm COM and COD crystals. CaOx crystals with size of 50 and 100 nm were transported to different regions in the cell and attacked the nucleus, which cause serious cell damage because the crystals can bypass lysosomes through the caveola-mediated pathway^31^. The uptake of the crystals with size of 50 and 100 nm was partly inhibited by treatment with amiloride hydrochloride. This finding is attributed to aggregation of nanosized crystals in culture media into large-sized crystals and internalization via macropinocytosis.

For COM-1 μm and COD-1 μm, the number of cells that internalized crystals was significantly decreased by treatment with amiloride hydrochloride. The uptake of COM-1 μm decreased from 24.38% to 5.48% (*p* < 0.01), whereas COD-1 μm decreased from 13.12% to 2.70% (*p* < 0.01) compared with those in the control group. These results indicated that the uptake of COM-1 μm and COD-1 μm was mainly through macropinocytosis. COM and COD crystals (50 nm, 100 nm, and 1 μm) were internalized by Vero cells through multiple pathways. The aggregation of small-sized crystals and the fragmentation of large-sized crystals can affect the cellular uptake pathway; this finding is similar to that in most previous studies on size-dependent cellular uptake mechanism^29^,^30^,^32^.

### Confocal microscopy analysis of crystal adhesion and internalization

**Figure 4:**
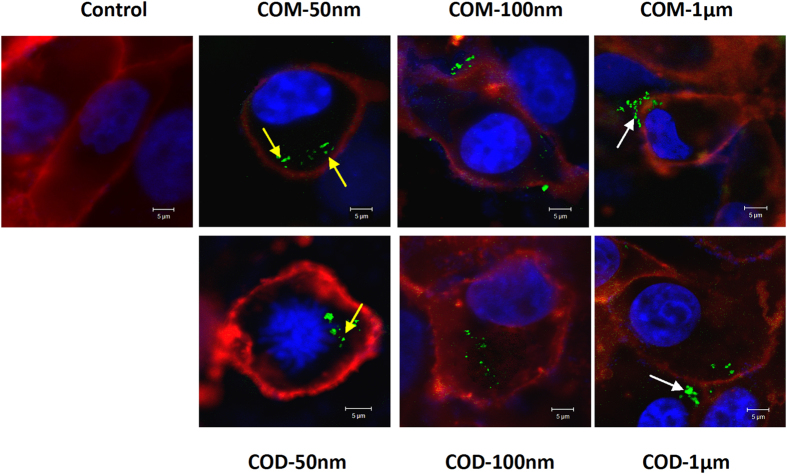
Confocal laser scanning microscopy of crystals internalized by Vero cells. Cells were treated with FITC-IgG labeled COM and COD crystals (green) for 6 h. Cell membrane was stained with DiI (red), and the nucleus was stained with DAPI (blue).

To verify crystal adhesion and internalization by Vero cells, we used confocal laser scanning microscopy to observe the intracellular and extracellular distribution of crystals. Crystals were labeled with FITC–IgG (green), whereas cell membrane was stained with DiI to present red fluorescence^33^. The nucleus was stained with DAPI (blue) to locate cell position. The results showed that control cells exhibited a typical spindle shape with full and intact morphology, whereas cells in crystal-treated groups showed morphological disorders and shrinking nuclei. Moreover, cells showed evident apoptosis in COM-50 nm and COD-50 nm treatment groups (Fig. 4). Nano-/micron-sized COM and COD crystals were internalized by Vero cells, and the internalization degree is higher in COM-50 nm and COD-50 nm than that in the other treatment groups (indicated by yellow arrow in Fig. 4). Adherent COM-1 μm and COD-1 μm were also observed on the membrane (indicated by white arrow in Fig. 4).

### Analysis of CaOx localization in lysosomes

COM and COD crystals were labeled with FITC–IgG (green). The lysosomes were then stained with Lyso-Tracker Red (red) to analyze the localization of nano-/micron-sized COM and COD crystals in subcellular compartments.

**Figure 5:**
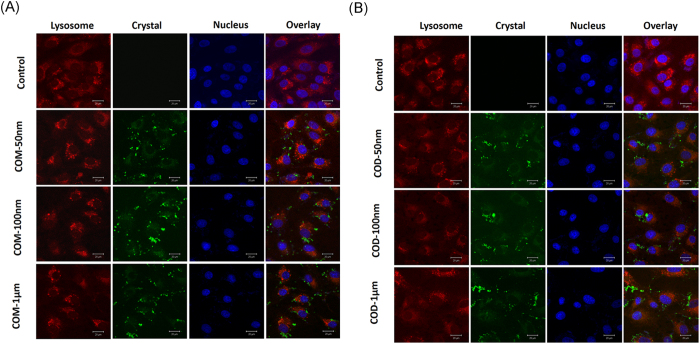
Distribution of the nano-/micron-sized crystals in Vero cells after exposure for 6 h. (**A**) COM treatment group and (**B**) COD treatment group. Lysosomes (red), nucleus (blue), and crystals (green) were labeled with Lyso-Tracker Red, DAPI, and FITC-IgG, respectively.

After incubating Vero cells and nano-/micron-sized crystals for 6 h, green dots were observed inside the cells, particularly in the lysosome (Fig. 5A and B). Hence, the endocytosis of crystals mainly involved lysosomes. Small crystal size led to large number of internalized crystals (Fig. 3; green fluorescence), which mainly merged with lysosomes (red fluorescence); as such, small-sized crystals were primarily distributed in lysosomes. Large crystal size led to reduced number of internalized crystals (Fig. 3), several internalized crystals were distributed in lysosomes, and most of the crystals adhered to the cell surface.

Figure 3 shows that the small-sized COM and COD crystals (50 and 100 nm) were mainly internalized through clathrin-mediated endocytosis. Small-sized crystals were coated with clathrin and transferred into endosomes through membrane ruffle or invagination. Early endosomes became late endosomes and were absorbed by lysosomes. Finally, the crystals were degraded by the lysosomes^34^. COM-1 μm and COD-1 μm crystals were internalized via the macropinocytosis pathway. The internalized crystals were coated into endocytic vesicles, dissolved into small particles, and degraded by lysosomes^35^.

### Lysosomal integrity

Acridine orange (AO), a metachromatic fluoro-probe, is a lysosomotropic component that accumulates in lysosomes by proton trapping. AO accumulation changes the fluorescence emission from green in the cytoplasm to red in the lysosomes^36^. Therefore, AO was used to determine lysosomal integrity by measuring the ratio of red and green fluorescence^37^. A low intensity of red fluorescence confers serious damage in lysosomes.

**Figure 6:**
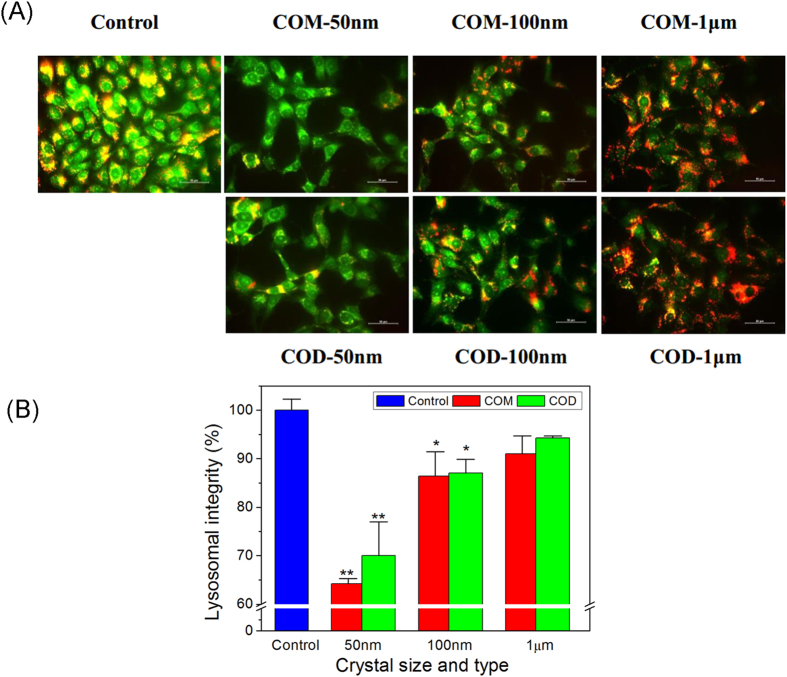
Lysosomal integrity after exposure to 200 μg/mL nano-/micron-sized COM and COD crystals for 6 h. (**A**) Fluorescence microscope observation and (**B**) quantitative analysis of lysosomal integrity in Vero cells. Red fluorescence represents complete lysosomes. Compared with the control group, *p < 0.05, **p < 0.01.

The extent of lysosomal integrity in Vero cells was visualized through fluorescence microscopy (Fig. 6A) and quantitatively analyzed using a microplate reader (Fig. 6B). AO-loaded lysosomes were uniformly distributed inside Vero cells in the control group. The emitted red fluorescence of lysosomes merged with the green fluorescence of the cytoplasm, thereby presenting orange fluorescence. However, crystals with size of 50 and 100 nm disrupted the distribution of AO in the cell. Most red fluorescence disappeared, but green fluorescence remained in the cell. This result indicated that the lysosomes were severely damaged, and AO that accumulated in the lysosome was released into the cytoplasm and presented green fluorescence. Cells in COM-50 nm and COD-50 nm treatment groups presented weak red fluorescence (Fig. 6B), which indicated that crystals severely damaged the lysosomes and disturbed AO inside the cells. The integrities of lysosomes decreased to 64.26% ± 1.04% (*p* < 0.01) and 70.05% ± 9.91% (*p* < 0.01) compared with those in the control group (100%), respectively.

Large-sized crystals (COM-1 μm and COD-1 μm) minimally influenced lysosomes. COM-1 μm and COD-1 μm caused cell shrinkage and cytoplasm condensation, but the red fluorescence, which represents complete lysosomes remained in the cells. In these treatments, the corresponding integrities of the lysosomes were 90.99% ± 3.70% (*p* > 0.05) and 94.31% ± 0.40% (*p* > 0.05) compared with those in the control group (100%), respectively. The ability of the four kinds of crystals to cause lysosome injury followed the order of COM-50 nm > COD-50 nm > COM-100 nm > COD-100 nm > COM-1 μm > COD-1 μm.

Lysosome is an important cell organelle that contains dozens of hydrolytic enzymes and resolves exogenous substance. The pH of lysosome is approximately 4.5 (intracellular pH 6.8–7.0)^38^. COM and COD crystals were internalized into the cell and then transported to the lysosomes. COM and COD crystals in the lysosomes were further degraded to oxalate and calcium ions. The released ions induced changes in the ionic strength inside the lysosomes, osmotic pressure imbalance, and disrupt lysosomal integrity^39^. Lysosomal damage can lead to cell necrosis and release of cathepsin, thereby inducing cell apoptosis^40^,^41^.

### Correlation of adhesion and internalization of CaOx with cell death

**Figure 7:**
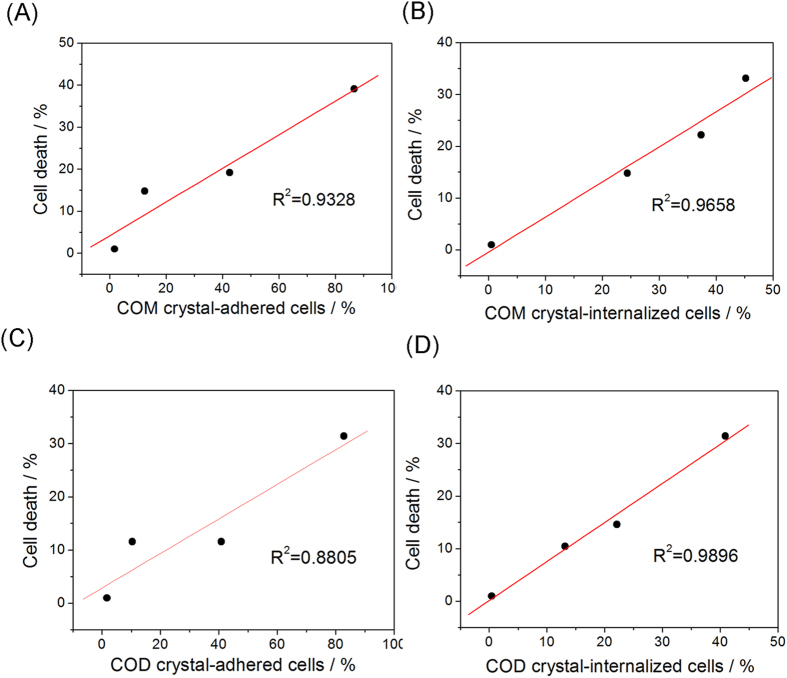
Correlation of adhesion and internalization of CaOx with cell death rate. R^2^ is the linear correlation coefficient.

Figure 7 illustrates the correlation of adhesion and internalization of CaOx with cell death after incubation of nano-/micron-sized crystals and Vero cells for 6 h. Cell death rate was determined by CCK-8 assay, and the proportion of cells that adhered or internalized crystals were determined from Figs 2 and 3. Figure 7 shows that the cell death rate was positively related to the amount of adhered or internalized crystals. The cell death rate increased with increasing amount of adhered or internalized crystals. Internalization as more closely related to cell death compared with crystal adhesion. For example, the coefficient of correlation (R^2^) for internalization was 0.9658, whereas that for adhesion was 0.9328 in the COM-treated group, by contrast, the coefficients in the COD-treated group were 0.9896 and 0.8805 for internalization and adhesion, respectively. Hovda *et al*.^6^ also demonstrated the correlation of the adhesion and internalization of CaOx crystals by kidney cells with cell death.

The crystal–cell interaction process (adhesion and internalization) produces numerous intracellular signaling events to regulate cell function and status^42^,^43^. Adhesion and internalization disrupt the cell membrane and disorganize the cell cytoskeleton^44^. Internalization of CaOx by cells into the cytoplasm would allow for direct interactions with intracellular organelles, resulting in cell death^37^,^45^. The correlation results showed that the internalization is a key process to cell death (Fig. 7); internalized crystals directly attack cell organelles^45^,^46^ and cause serious and direct damages. For example, COM reduces the mitochondrial membrane potential and induces irreversible apoptosis^47^. COM crystals inhibited mitochondrial respiration, resulting in changes in mitochondrial permeability, dysfunction of mitochondrial function, and collapse of cellular ATP-induced cell death^48^. However, the lysosomes need to secrete excess hydrolytic enzymes to degrade the internalized CaOx crystals. Therefore, the internalized COM increased the burden of the lysosomes, and lysosomal damage caused cell death^49^.

### Cellular internalization mechanism of nano-/micron-sized CaOx crystals

**Figure 8:**
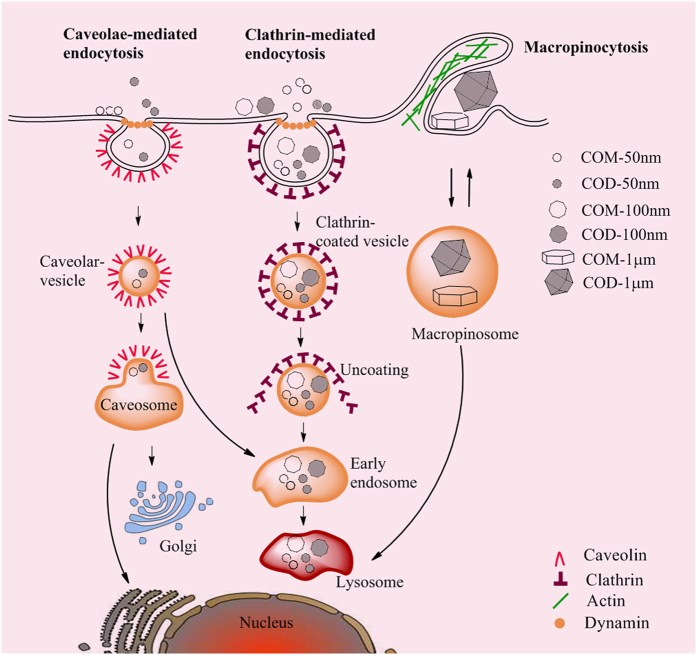
Schematic of COM and COD crystals on cellular internalization pathways in Vero cells.

Basing on the experimental results (Figs 2, 3, 4, 5, 6 and 7), we proposed a schematic illustration of the cellular internalization mechanism of Vero cells after exposure to nano-/micron-sized CaOx crystals (Fig. 8). The cellular internalization of CaOx crystals toward Vero cells is size dependent. Nanosized crystals (50 and 100 nm) preferred to be internalized via the clathrin-mediated pathway. COM and COD were first enclosed within clathrin-coated vesicles derived from folds or invaginations of the plasma membrane. These crystals were brought into cells and became endosomes; early endosomes became late endosomes and captured by lysosomes. Finally, the crystals were degraded by lysosomes in low-pH environment (Fig. 5). This process increased the burden of the lysosomes and the large range of lysosomal rupture (Fig. 6) ultimately led to cell death. Meanwhile, low amounts of COM and COD crystals with size of 50 and 100 nm were internalized through caveola-mediated endocytosis. Crystals were coated by caveolae within milder pH and biological condition and then transported to the endoplasmic reticulum and Golgi complex. In this process, crystals could bypass lysosome transport to other parts of the cell as well as entry and attack to the nucleus. These characteristics are key factors affecting cell death caused by COM and COD with size of 50 and 100 nm (Fig. 7). Vero cells mainly internalized 1 μm sized crystals through macropinocytosis. The crystals triggered the formation of membrane ruffles, which protruded to engulf the surrounding crystals. These crystals simply melt with the cell membrane or form intracellular vacuoles, which are degraded by lysosomes or excreted from cells. In this process, COM-1 μm and COD-1 μm caused mild cell damage.

## Conclusions

The cytotoxicity, adhesion, and internalization of COM and COD crystals with different sizes were explored in Vero cells. The adhesion and internalization of COM and COD crystals toward Vero cells were closely related to the size of the crystals. Crystals with small sizes exhibited enhanced adhesion and internalization as well as high cell death rate. Nanosized (50 and 100 nm) CaOx crystals preferred to be internalized via clathrin-mediated pathways, whereas micron-sized (1 μm) crystals favored to be internalized through macropinocytosis. The internalized crystals were mainly distributed in the lysosomes and destroyed the integrity of the lysosomes to some extent. The internalization pathway of CaOx crystals is related to crystal size but not to crystal phase. This study provides a basis for tracking endocytosed crystals inside the renal tubular cells during kidney stone formation and for developing potential medicine for inhibiting crystal uptake, cell injury, and stone formation.

## Materials and Methods

### Materials and apparatus

African green monkey renal epithelial (Vero) cells were purchased from Shanghai Cell Bank, Chinese Academy of Sciences (Shanghai, China). Dulbecco’s modified eagle medium (DMEM), fetal bovine serum, penicillin and streptomycin were obtained from HyClone Biochemical Products Co., Ltd. (UT, USA). Cell culture plates were purchased from Wuxi Nest Bio-Tech Co., Ltd. (Wuxi, China).

Rabbit anti-mouse IgG conjugated with fluorescein isothiocyanate (FITC-IgG), Lyso-Tracker Red, DiI (1,1′-dioctadecyl-3,3,3′,3′-tetramethylindocarbocyanine perchlorate DiIC18(3)), anti-fade fluorescence mounting medium were all purchased from Shanghai Beyotime Bio-Tech Co., Ltd. (Shanghai, China). Acridine orange (AO) was purchased from Sigma-Aldrich (St. Louis, MO, USA). Sucrose, methyl-β-cyclodextrin and amiloride hydrochloride were purchased from Aladdin-reagent Co., Ltd. (Shanghai, China). Calcium chloride (CaCl_2_), sodium oxalate (Na_2_Ox), paraformaldehyde, ethanol were all analytical pure and purchased from Guangzhou Chemical Reagent Factory of China (Guangzhou, China).

X-L type environmental SEM (Philips, Eindhoven, Netherlands); confocal laser scanning microscope (LSM510 Meta Duo Scan, Zeiss, Germany); microplate reader (Safire2, Tecan, Männedorf, Switzerland); flow cytometer (FACS Aria, BD Corporation, Franklin Lakes, NJ, USA); fluorescence microscope (Leica DMRA2, Germany).

### Preparation of fluorescence-labeled calcium oxalate crystals

Different-sized COM and COD crystals (50 nm, 100 nm, and 1 μm) were prepared as previously described^18^, the size and crystalline phase of prepared crystals were characterized by SEM, XRD and FT-IR.

Preparation of FITC-IgG fluorescence-labeled crystals: Briefly, 10 μL of rabbit anti-mouse IgG conjugated with FITC (1 mg/mL) was mixed with 4 mL of CaOx crystals (400 μg/mL). The mixture was incubated at 37 °C overnight in the dark. Free FITC-IgG was removed using a dialysis bag (M_w_8000–14,000, Sigma, USA) for 48 h. Fluorescence-labeled CaOx crystals were harvested, washed, and dried. The crystals were then suspended in anhydrous ethanol by ultrasonic dispersion for 10 min and dropped on the slide before fluorescence microscopy detection. Percentages of fluorescent-labeled COM and COD crystals were detected by flow cytometry.

### Cell culture and exposure to COM and COD crystals

Vero cells were cultured in DMEM containing 10% fetal bovine serum and 100 U/mL penicillin–100 μg/mL streptomycin with pH 7.4 at 37 °C in 5% CO_2_ humidified environment. Upon reaching 80–90% confluent monolayer, the cells were blown gently after trypsin digestion to form cell suspension. For experiments, the cells were seeded in culture plates at a density of 1 × 10^5^ cells/mL and allowed to attach for 24 h. The cells were then treated with varying sizes of COM and COD crystals suspended in DMEM (200 μg/mL) for a certain time. Cells maintained in DMEM without COM and COD crystals were used as control group.

### Quantitative analysis of adherent calcium oxalate crystals by flow cytometry

Vero cells were cultured at 4 °C for 30 min to block cell endocytosis, at which crystals only adhered to the cell surface. The cells were exposed to three sizes of fluorescence-labeled COM and COD crystals (50 nm, 100 nm, and 1 μm) at a concentration of 200 μg/mL and incubated at 4 °C for 1 h. Afterward, the cells were washed twice with cold PBS (to eliminate the unbound crystals), followed by trypsinization. The cells were resuspended with 200 μL of PBS. The percentage of cells with the adhered crystals was quantitatively determined by flow cytometry analysis. Cells with positive FITC signal were directly counted and considered as containing adherent crystals.

### Quantitative analysis of internalized calcium oxalate crystals by flow cytometry

The experimental model was divided into four groups: 1) control group, in which only normal serum-free culture medium was added and incubated for 6 h; 2) normal endocytosis group, in which cells were exposed to three sizes of fluorescence-labeled COM and COD crystals (50 nm, 100 nm, and 1 μm) at a concentration of 200 μg/mL for 6 h at 37 °C; 3) low temperature endocytosis group, the cells were treated with fluorescence-labeled COM and COD (200 μg/mL) for 6 h at 4 °C for the energy-dependent endocytosis; 4) endocytosis inhibitor group, the cells were preincubated with different endocytosis inhibitors (sucrose, methyl-β-cyclodextrinc and amiloride hydrochloride) for 30 min and washed three times with PBS according to the method reported previously^8^,^9^. The cells were treated with fluorescence-labeled COM and COD (200 μg/mL) for 6 h and rinsed with PBS (to eliminate the unbound crystals). According to the method from literature^6^, the adherent COM crystals were dissolved by 5 mM EDTA in PBS for 5 min. Free FITC-IgG was washed by PBS. The cells were resuspended with 200 μL of PBS. The percentage of cells with internalized crystals was quantitatively determined by flow cytometry analysis. Cells with positive FITC signal were directly counted and regarded as internalized crystals.

### Confocal microscopy observation of intracellular crystal distribution

Cells were incubated with fluorescence-labeled COM and COD crystals (50 nm, 100 nm, and 1 μm) at a concentration of 200 μg/mL for 6 h. The cell membrane was stained with DiI (10 μM, 300 μL) for 10 min, and then the cells were fixed with paraformaldehyde (4%) for 30 min in PBS. DAPI staining solution was then added to the cells and incubated for 5 min. The cells were again washed three times with PBS for 5 min. Finally, the prepared samples were observed in a confocal microscope.

### Accumulation of calcium oxalate within lysosome

Cells were treated with fluorescence-labeled COM and COD (50 nm, 100 nm, and 1 μm) at a concentration of 200 μg/mL for 6 h. The cells were stained with 70 nM Lyso-Tracker Red to label lysosomes for 2 h and then fixed with 4% paraformaldehyde for 10 min, the cell nucleus were stained with DAPI. Accumulation of calcium oxalate crystals with lysosome were observed in confocal laser scanning microscope.

### Lysosomal integrity assay

For fluorescence qualitative observation by fluorescence microscope, the cell suspension (1 mL) with a cell concentration of 1 × 10^5^ cells/mL was inoculated to sub-confluence in 12-well plates with coverslips for 24 h. The cells were washed twice with PBS, and then loaded with 5 μg/mL AO in DMEM for 15 min. Subsequently, the cells were incubated in serum-free culture media with 200 μg/mL nano-/micron-sized COM and COD crystals for 6 h.

For fluorescence quantitative detection by microplate reader, cells (1.0 × 10^5^ cells/ml) were cultured in a 96-well plate (100 μL/well) and were stained with AO, the cells were washed with PBS before fluorescence measurements with excitation at 485 nm and emission at 530 (green cytoplasmic AO) and 620 nm (red lysosomal AO). Normal lysosomal integrity = (total red fluorescence intensity of normal lysosome)/(total green fluorescence intensity of normal lysosome). Lysosomal integrity = (total red fluorescence intensity)/[(total green fluorescence intensity) × (normal lysosomal integrity)].

### Effect of calcium oxalate crystals on cell viability

Cells were treated with COM and COD (50 nm, 100 nm, and 1 μm) at a concentration of 200 μg/mL for 6 h. Then 10 μL CCK-8 was added to each well and incubated for 1.5 h in an incubator at 37 °C. Absorbance (A) was measured by microplate reader at 450 nm. The correlation between cell viability and crystal-adhered or crystal-internalized cells were analyzed. Cell viability was determined using the equation below.


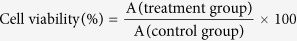


### Statistical method

The experimental results were analyzed statistically in SPSS 13.0 (SPSS Inc., Chicago, IL, USA) and expressed as mean ± SD from three independent experiments. For analysis of the amount of adherent crystals and lysosomal integrity, we performed the Paired-Sample T Test. For quantitative analysis of the amount of internalized crystals, we performed one-way ANOVA with Tukey post-test. If *p* < 0.05, there was significant difference; if *p* < 0.01, the difference was extremely significant; if *p* > 0.05, there was no significant difference.

## Additional Information

**How to cite this article**: Sun, X.-Y. *et al*. Size-dependent cellular uptake mechanism and cytotoxicity toward calcium oxalate on Vero cells. *Sci. Rep.* **7**, 41949; doi: 10.1038/srep41949 (2017).

**Publisher's note:** Springer Nature remains neutral with regard to jurisdictional claims in published maps and institutional affiliations.
